# Mons. L. Baudens

**Published:** 1858-05

**Authors:** 


MILITARY surgery in France has lately sustained a severe loss in the death of MONS. L. BAUDENS, which took place at Paris on the 27th of January last, in consequence of cancer of the liver. He was the author of an excellent work on Gunshot Wounds, published in 1836; served with great distinction in the Crimean war, and held, at the time of his demise, the office of one of the chief Medical Inspectors of the French army.

